# Generalized conditions of spherical carbonate concretion formation around decaying organic matter in early diagenesis

**DOI:** 10.1038/s41598-018-24205-5

**Published:** 2018-04-20

**Authors:** Hidekazu Yoshida, Koshi Yamamoto, Masayo Minami, Nagayoshi Katsuta, Sirono Sin-ichi, Richard Metcalfe

**Affiliations:** 1 Material Research Section, Nagoya University, University Museum, Chikusa, Nagoya, Japan; 2 Graduate School of Environmental Studies, Nagoya University, Chikusa, Nagoya, Japan; 3 Institute for Space-Earth Environmental Research, Nagoya University, Chikusa, Nagoya, Japan; 4 Faculty of Education, Gifu University, Gifu, Japan; 5 Quintessa Limited, The Hub, Henley-on-Thames, Oxfordshire, UK

## Abstract

Isolated spherical carbonate concretions observed in marine sediments are fascinating natural *objet trouve* because of their rounded shapes and distinct sharp boundaries. They occur in varied matrices and often contain well preserved fossils. The formation process of such concretions has been explained by diffusion and rapid syn-depositional reactions with organic solutes and other pore water constituents. However, the rates, conditions and formation process of syngenetic spherical concretions are still not fully clear. Based on the examination of different kinds of spherical concretions from several locations in Japan, a diffusion based growth diagram was applied to define the generalized growth conditions of spherical concretions formed around decaying organic matter. All analytical data imply that the spherical concretions formed very rapidly, at least three to four orders of magnitude faster than previously estimated timescales. The values indicate that spherical concretions are preferentially grown within clay- to silt-grade marine sediments deposited in relatively deep (a few tens of metres) environments dominated by diffusive solute transport, very early in diagenesis.

## Introduction

Spherical, isolated carbonate concretions occur throughout the world in marine argillaceous sedimentary rocks of widely varying geological ages. These concretions are characteristically highly enriched in CaCO_3_ compared to the surrounding sedimentary rock matrices and are typically separated from these rock matrices by sharp boundaries^1–3^. These sharp boundaries mean that the isolated carbonate concretions are readily identifiable and both geologists and non-scientists alike have been motivated to consider how such concretions could have formed.

It is also known that many of these concretions have various kinds of well-preserved fossils at their centres^3^. Isotopic analyses have been used to identify the source of carbon that formed the concretions^4–6^ and to understand the diagenetic processes, including microbial activity, that occurred during sediment burial and concretion formation^1,7,8^. However, even though there have been many studies of concretions over several decades, there still remain questions regarding concretion formation^9–11^. The remaining questions include: How rapidly do concretions grow? Why are the concretions often spherical with sharp boundaries? and Why does the localization of Ca and CO_3_, and consequently concretion growth, stop? These questions can be answered by determining the relationship between the formation conditions of the spherical concretions, the mass transport processes in the sedimentary matrix, and the concretion growth rates. However, this relationship has yet to be described precisely.

**Figure 1:**
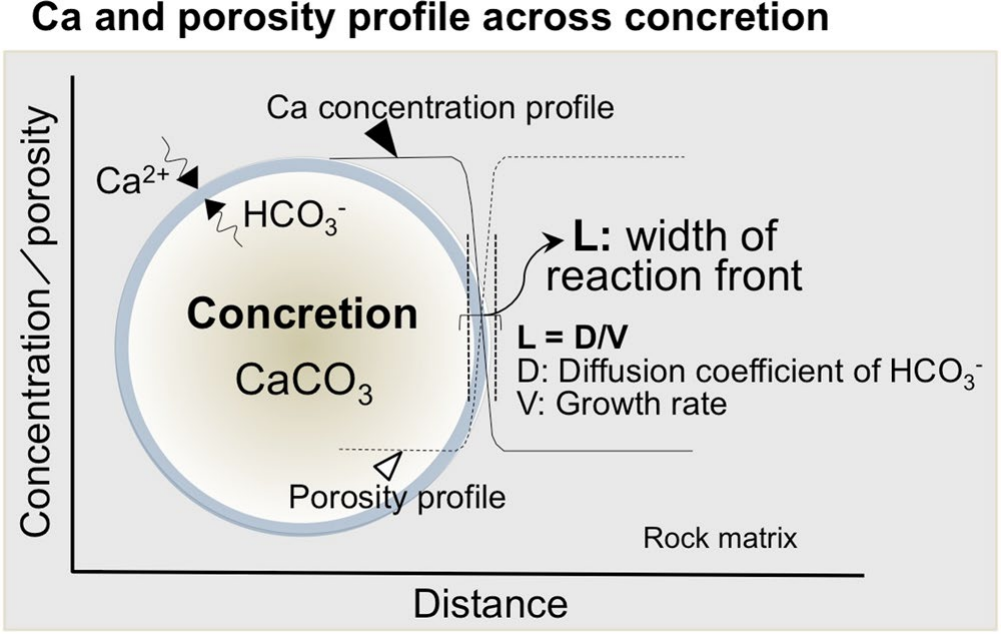
Conceptual view of the features of spherical concretions. Ca profile and porosity distribution across the spherical concretion forming under rather stable conditions under which solutes diffused continuously, causing calcite precipitation until the organic source of carbon at the centre of the concretion was consumed.

For several decades, the formation of spherical concretions has been explained by diffusion accompanied by carbonate inter-conversion reactions^12,13^ and also by very slow three dimensional advection of water when cementation conditions are isotropic^9,14^. These processes give rise to a spherical morphology in homogeneous sediments or sedimentary rocks. The process is basically accepted, but previously proposed models cannot explain the steep chemical gradients (notably of Ca) that occur across the margins of almost all concretions, and also the constant calcite (CaCO_3_) concentration^3,15^ and constant porosity within the body of a concretion^16,17^, as determined by thin section analysis and porosity measurements^3^. Such a steep boundary occurs as the reaction front on the concretion surface (Fig. 1) is developed by reactions between HCO_3_^−^ and Ca^2+^ ions as concretions grow outwards, and has a certain characteristic width ‘L’ where CaCO_3_ has been precipitated. This front is developed in any kind of carbonate-rich spherical concretion formed syn-genetically during burial of marine sediments with organic carbon sources in the concretions^3,18,19^. In this case, the relationship between ‘L’, diffusion coefficient ‘D’ of HCO_3_^−^ and growth rate of the concretion ‘V’ is: ‘L = D/V’. This relationship can be illustrated using a ‘Diffusion-growth rate cross-plot’, which was developed to explain detailed observations on concretions at a single locality in Japan^3^. Here, we extend the theory of the ‘cross-plot diagram’ by applying it to concretions of different ages at several localities to develop a general model for the formation conditions of spherical carbonate concretions in marine sediments. The outcome of the analysis is also used to answer the above remaining questions about syn-genetically formed spherical carbonate concretions and to build on the previous study, which only investigated concretions from a single locality.

**Figure 2:**
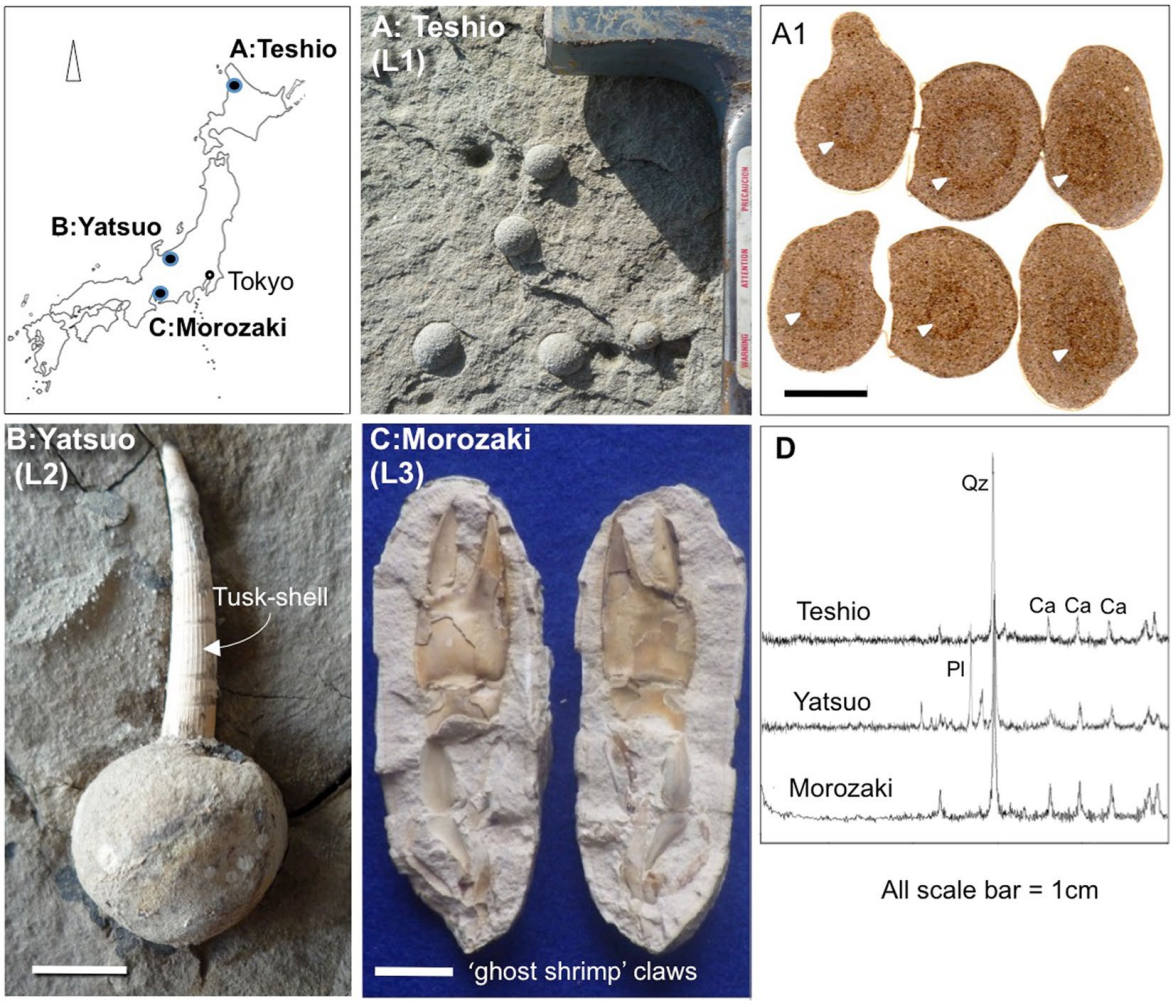
Sampling locations and the characteristics in hand specimen of studied Japanese spherical carbonate concretions. (**A**) Teshio concretions (L1); (**B**) Yatsuo tusk-shell concretions (L2), and (**C**) Morozaki ‘ghost-shrimp’ concretions (L3). Teshio concretions from Hokkaido with rounded impressions in their centers suggesting the presence of a non-skeletal animal (arrows: A1). δ^13^C data also suggest an organic carbon origin. (**D**) X-ray diffractograms for each concretion shows a clear calcite peak. Index map is based on the data of Geospatial Information Authority of Japan website (http://www.gsi.go.jp/maps.gsi.go.jp). All photographs (**A**–**C**) shown here are taken by H.Yoshida.

The widths of reaction fronts (L) at the margins of spherical carbonate concretions of various sizes were measured to determine the relationship between ‘L’ and concretion diameter (R). The widths ‘L’ of three different kinds of spherical concretions from Japan were determined by using an X-ray analytical microscopy (SXAM) method to determine the Ca distribution and concentration profile between the body of each concretion and the surrounding clay to silt matrix. The examined calcium carbonate concretions are Teshio concretions (numbers of analysed samples, n = 20) from Cretaceous sediments, Yatsuo tusk-shell concretions (n = 24) and Morozaki ‘ghost-shrimp’ concretions (n = 10) from Oligocene sediments (Fig. 2) (Supplementary Table 1). The host sediments of the spherical carbonate concretions at these three localities are all of marine origin and are composed of clay to silty facies composed of very fine grains. The characteristics of these facies are consistent with a relatively deep seabed depositional environment (a few tens of metres^20–23^), without rapid flow of water.

The Teshio concretions are found in northern Hokkaido close to Wakkanai city in Late Cretaceous clay to silty sedimentary rocks of the Saku Formation^24^. This formation belongs to the Ezo Group^20^, which is distributed in and around the northern part of Hokkaido. The concretions occur mainly in silty facies and are distinguished by their spherical shape with diameters of ca. 1 to 2 cm (Fig. 2A). The fabric shows bending of sedimentary laminae around the concretions due to compaction. These concretions contain circular patterns at their centres, suggesting the past presence of non-skeletal organic material (Fig. 2A-1).

Yatsuo tusk-shell concretions are found in the Kurosedani Formation of the Yatsuo Group, which is distributed in Toyama Prefecture, central Japan (Fig. 2B). The sizes of the concretions vary from 1.5 to 3 cm. All tusk-shell concretions are formed around the mouths of shells situated in the concretions’ centres^3^. The Kurosedani Formation consists of argillaceous sedimentary rocks that have been determined to be of Miocene age (17–15 Ma) using diatoms and planktonic foraminifera^21^. In outcrops the tusk-shells (*Fissidentalium* spp.)^22^ are seen to lie in the compacted clay matrix almost parallel to the microscopic sedimentary fabric. However, the very fine fabric around the concretions is bent due to compaction after concretion formation^3^.

The Morozaki ‘ghost-shrimp’ concretions are found in the Morozaki Formation, which is distributed in the Chita peninsula of Aichi prefecture, central Japan (Fig. 2C). The concretions are known to have formed around ‘ghost shrimp’ claws within tuffaceous and clayey sediments deposited in relatively deep (a few tens of metres) environments^23^, which based on diatom stratigraphy are estimated to have been deposited between 15–18 Ma^23^. All the concretions are formed around a central claw of a *Callianassa* spp.^25^ which is typically 3 to 7 cm across. Sedimentary fabrics are also bent around ‘ghost-shrimp’ concretions showing that the spherical concretions formed before consolidation and compaction of the sediment.

The relationship between the width of reaction front ‘L’, diffusion coefficient ‘D’ of HCO_3_^−^ and growth rate of the concretion ‘V’, L = D/V, suggests that the ‘L’ will increase almost linearly with increasing growth time and hence increasing diameter (R) of a concretion. In order to evaluate the general applicability of this relationship, other ‘L’ values for larger concretions, for example concretions from the Lower Lias of Dorset, southern England (where the concretions are known as ‘*Coinstones’*)^26^, ‘*Curling Stones’* from the Upper Lias, Lower Jurassic sediments exposed on the NE coast of Yorkshire, England^15^ and ‘*Moeraki boulders’* observed in the mudstone formation from New Zealand^27,28^ were also estimated based on reported CaCO_3_ contents.

## Results

**Figure 3:**
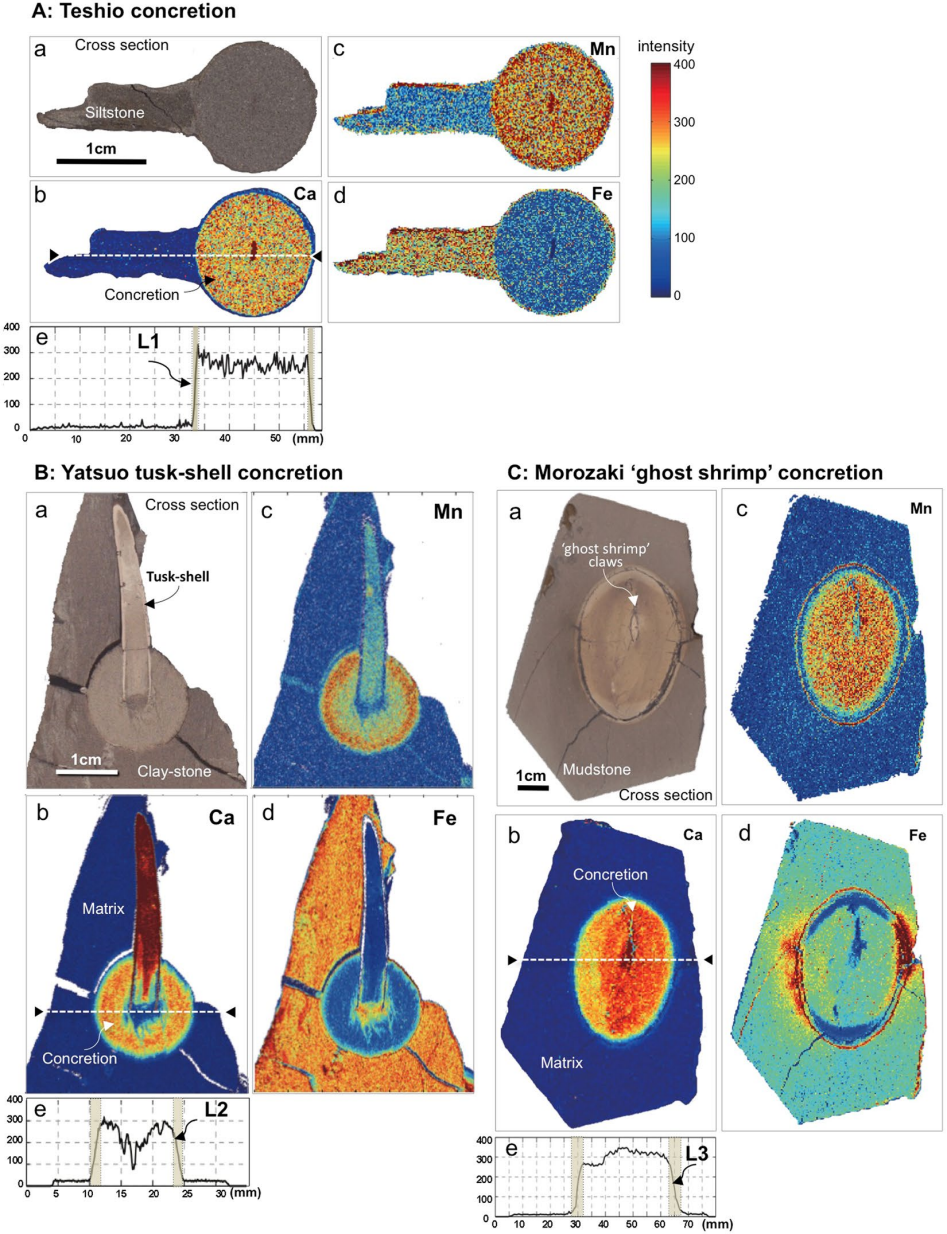
SXAM mapping and ‘L’ of concretions. The reaction front ‘L’ identified by SXAM mapping of syn-genetically formed concretions (**A**; Teshio, **B**; Yatsuo and **C**; Morozaki). (**A**) Ca (b), Mn (c), Fe (d) distributions and L1 (e) defined by Ca profile. Teshio concretions have diameters of 1~2 cm and very thin reaction fronts (L1) of 1 mm. (**B**) Ca (b), Mn (c), Fe (d) distributions and L2 (e) defined by Ca profile. Tusk-shell (*Fissidentalium* spp.) concretions with a diameter of up to 1.5~3 cm and reaction fronts (L2) a few mm wide. (**C**) Ca (b), Mn (c), Fe (d) distributions and L3 (e) defined by Ca profile. Morozaki ‘ghost shrimp’ concretions formed around *Callianassa* spp. have diameters of 3~7 cm with reaction fronts of 3~6 mm (L3). SXAM Ca X-ray intensity in and around the cut surface of a concretion shows a sharp boundary ‘L’ between the concretion and matrix defined by Ca concentration gradients. Colour bar shows X-ray intensity of all measured elements. All photographs (**A**–**C**) shown here are taken by H.Yoshida.

All concretions have sharp boundaries between the cemented bodies and surrounding matrices, allowing their spherical shapes to be seen readily at outcrop (Fig. 2). All the kinds of concretions examined were determined by X-ray diffraction to be composed of calcite (Fig. 2D). With these concretions, the widths of reaction fronts ‘L’ of the Teshio, Yatsuo and Morozaki concretions, as defined by the Ca profiles and the distributions of Fe and Mn analysed by SXAM, are shown in Fig. 3A–C. Ca concentrations were found to be similar across a broad width of a concretion, but decreased rapidly across the rim of the concretion. Fe and Mn concentrations vary inversely with respect to one another. The profiles of all concretions show that the Ca concentrations are almost flat inside a concretion then tail to the concentrations in the surrounding rock matrix across a thin layer with width ‘L’. The profiles also show that Fe concentrations are elevated in the volume originally occupied by an organism, but Mn concentrations are relatively low in this zone. Outside this zone, Fe concentrations are relatively low, but Mn concentrations increase towards the margin of the concretion.

Chemical compositions of the spherical concretions measured by XRF are shown in Table 1. All concretions have a high concentration of Ca, from several times to 20 times higher than surrounding matrices. P is also remarkably concentrated in the concretions, while the content of Si is lower. Almost the same variations in elemental concentrations are observed in the larger concretions, ‘*Coinstones*’^26^, ‘*Curling Stones*’^15^ and ‘*Moeraki boulders*’^28^, from sites outside Japan.

**Table 1:** Results of XRF analysis of examined concretions.

	Teshio				Yatsuo				Morozaki			
	Concretion			matrix	Concretion			matrix	Concretion			matrix
	1	2	3	4	5	6	7	8	9	10	11	12
SiO_2_	39.48	35.36	35.60	54.00	28.79	31.64	28.05	62.54	15.16	14.98	17.99	62.92
TiO_2_	0.31	0.26	0.26	0.45	0.25	0.29	0.24	0.92	0.13	0.13	0.15	0.51
Al_2_O_3_	9.41	8.90	8.95	12.77	6.68	7.43	6.46	17.79	3.54	3.48	4.15	12.83
Fe_2_O_3_	2.92	2.82	2.70	2.96	2.21	2.48	2.15	6.90	1.93	1.69	1.94	4.10
MnO	0.05	0.03	0.03	0.28	0.63	0.43	0.69	0.03	0.07	0.08	0.08	0.02
MgO	0.78	0.72	0.76	0.90	2.93	3.08	2.85	2.43	2.30	2.61	2.05	1.47
CaO	23.18	26.02	25.74	11.63	51.67	48.34	52.12	3.99	39.83	41.27	36.07	1.30
Na_2_O	2.16	1.92	1.94	2.36	0.70	0.81	0.69	1.86	0.79	0.78	0.95	3.14
K_2_O	1.42	1.33	1.37	2.40	0.67	0.80	0.64	1.79	0.41	0.36	0.55	0.08
P_2_O_5_	0.18	0.19	0.18	0.12	2.39	1.15	2.96	0.13	1.48	1.59	1.44	0.08
Ig.Loss(wt%)	18.58	20.89	20.75	9.32	32.34	30.63	31.51	12.12	33.70	31.84	34.26	6.26
Total (wt%)	98.44	98.40	98.25	97.17	96.91	97.07	96.45	98.38	98.85	98.3	99.13	95.63

SXAM mapping and XRF measurements clearly show that all concretions measured have quite similar reaction front widths, ‘L’, readily defined by a steep change of Ca concentration, from a higher and almost constant concentration within the concretion (CaO: 30~60 wt%) to a lower background level (CaO: 1~12 wt%) into the matrix. Based on the measured Ca profiles across the concretions, ‘L’ varies as follows. The smallest concretions i.e. Teshio concretions, (Fig. 3A) give Ca profiles that indicate ‘L’ of about 1 mm for concretions with diameters of 1 to 2 cm. Yatuso tusk-shells concretions (Fig. 3B) have diameters of 1.5 to 3 cm and give ‘L’ of 2 to 3 mm. The slightly larger Morozaki ‘ghost shrimp’ concretions (Fig. 3C) have diameters of 3 to 7 cm and wider ‘L’ of 3 to 6 mm.

δ^13^C_PDB_ data of all concretions are also shown in Table 2. The values of all measured concretions are relatively low (Teshio: −21.9~−15.6‰; Yatuso: −19.2~−15.9‰; Morozaki: −16.1 ~ −7.3‰) and the larger concretions described in published literature also have low δ^13^C (*Coinstone*: −17.5~−10.5‰; *Curling stones*: −15.4~−13.7‰; *Moeraki boulders*: −30.8~−15.2‰) suggesting an organic origin of the carbon^15,26,28^.

**Table 2:** Results of C content and δ^13^CvsPDB analysis of examined concretions.

			C (wt%)	δ^13^CvsPDB(‰)
Teshio	1	Concretion	4.79	−21.9
	2		5.38	−20.1
	3		4.45	−16.1
	4		6.61	−16.1
	5		4.47	−15.6
	6	matrix	1.56	−11.3
	7		0.03	−11.2
Yatsuo	1	Concretion	7.20	−19.2
	2		7.87	−19.1
	3		8.36	−18.3
	4		6.07	−15.9
	5		7.54	−18.7
	6		8.86	−19.0
	7		6.20	−17.6
	8	matrix	0.01	−6.4
	9		0.01	−7.6
	10		0.01	−8.3
Morozaki	1	Concretion	6.61	−16.1
	2		0.03	−11.2
	3		8.56	−7.3
	4	matrix	8.58	−1.2
	5		7.29	3.1
	6		0.06	0.03

The carbon source of concretions with lower δ^13^C values are consistent with the HCO_3_^−^ that precipitated as calcite being a byproduct of fatty acid (R-COOH) decomposition that originates in decaying organisms within concretions^3^. This suggests that the concretions have been formed by reaction between carbon supplied by this organic source and Ca^2+^ in the surrounding seawater-derived pore-water. Therefore, steep Ca concentration profiles cross the margin of a concretion must be controlled by the rates of diffusion of HCO_3_^−^, from inside towards the margin, and of Ca^2+^ from pore-water within the surrounding marine sediments towards the concretion. The HCO_3_^−^ has a higher concentration within the concretion than outside it, as well as a higher apparent diffusion coefficient than Ca^2+^ ^29^. The reaction front at the margin of the concretion is therefore characterised by rapid precipitation of CaCO_3_ due to super saturation and a pH increase at the cementation front^30^. The Fe and Mn profiles across the concretions could be explained by the conditions towards the margin of the concretion being less reducing and more alkaline than those near the decaying organism; the stability field of Mn-carbonate extends to higher pH and Eh than the stability field of Fe-carbonate^31^.

Although calcite (CaCO_3_) precipitated preferentially within the initially relatively large pores in the matrix, micro-pores within the concretion continued to function as migration paths for HCO_3_^−^ at the reaction front. Due to this, concentrations of both HCO_3_^−^ and Ca^2+^ in the pore-water were decreased within the reaction front and the changed concentration gradients drove further supply of Ca^2+^ to the reaction zone until the HCO_3_^−^ concentration decreased to a level controlled by CaCO_3_ equilibrium and all the organic carbon was consumed. The concretions therefore continue to grow until there is no more carbon of organic origin remaining within the concretion. Concretion formation then stops and the concentrations of Ca^2+^ in the pore-water at the reaction front remain at the levels of surrounding seawater-derived pore-water.

A tusk-shell concretion contains almost the same mass of C (Vc) as the mass of organic C estimated to have occurred initially in the cone shaped tusk-shell (Vt), based on the simple mass balance calculation below, using measured data^3^. This mass balance supports the conclusion that the C source was provided by decaying organic matter.

The mass of C within a tusk-shell spherical concretion is given by: Vc = 4/3πr_c_^3^ · d(c) · n_c_, where r_c_ is the radius of a concretion (mean value; 0.75 cm), d(c) is the average density of a concretion (1.7 g/cm^3^) and n_c_ (0.07) is the proportion of C in the concretion. Using the measured values for the various parameters gives Vc ≃ 0.20 g.

The mass of organic C within a tusk-shell can be estimated by treating a tusk shell as if it is a cone: Vt = 1/3πr_t_^2^ · l · d(o) · n_o_, where r_t_ is the radius of a tusk-shell’s mouth (mean value; 0.25 cm), l (6 cm) is the average length of a Yatsuo tusk-shell, d(o) is the density of living Crustacea (1.1~1.3 g/cm^3^)^32^ and n_o_ (0.4) is the proportion of organic C in the living tusk-shell. Using the measured values for the various parameters gives Vt ≃ 0.17~0.20 g, which is almost the same as calculated for Vc.

**Figure 4:**
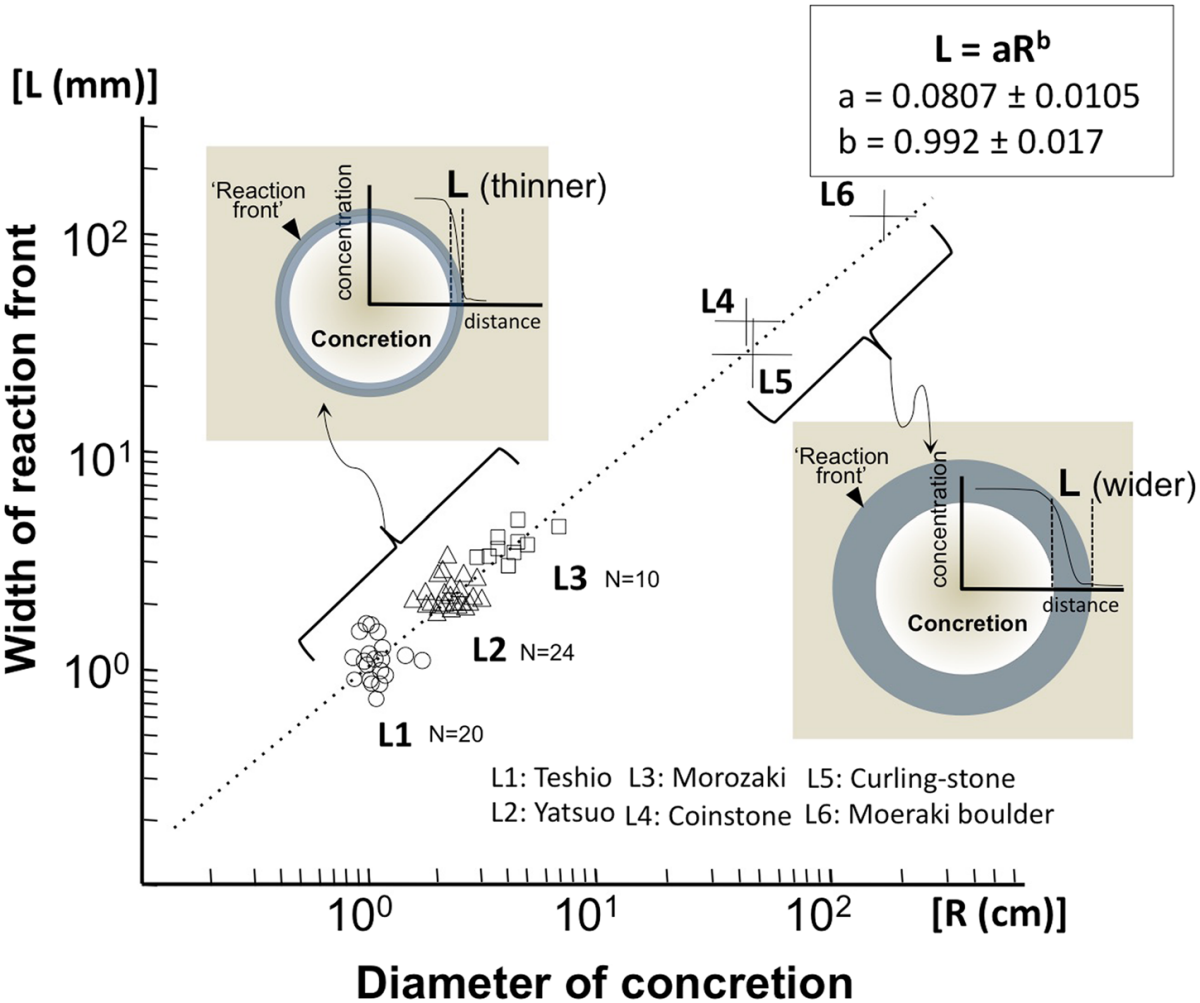
Relationship between concretion diameters (R) and widths of reaction fronts (L). The measured data from L1 to L3 and estimated value from L4 to L6 show an almost linear correlation between the width of reaction front ‘L’ and the diameter of the concretion ‘R’ suggesting that concretions were formed under relatively stable sedimentary environments controlled by diffusive solute migration, which is expressed as L = aR^b^, where a = 0.0807 ± 0.0105 and b = 0.992 ± 0.017.

As the organic matter decays diffusion is driven by the changing HCO_3_^−^ concentration gradient during the consumption of the organic carbon source. ‘L’ will be thinner at the beginning of concretion formation, but become wider as the carbon source is consumed and the concretion grows (hence larger concretions have greater ‘L’). The correlation between ‘L’ and ‘R’ can be clearly seen in Fig. 4 with measured values (L1~L3) and the estimated values (L4~L6) of larger concretions (Supplementary Table 1 and Figs 1 and 2).

The least squares fitting of Fig. 4 gives L = aR^b^, where a = 0.0807 ± 0.0105 and b = 0.992 ± 0.017. It can be seen that there is a strong positive correlation between the thickness L and the diameter of a concretion R. This correlation can be explained as follows. CaCO_3_ molecules precipitate through reaction of Ca^2+^ and CO_3_^2−^. If the rate of precipitation is high, the concentration of Ca^2+^ ion at the concretion surface is very low. Assuming the concentration of Ca^2+^ ion is quite low at the surface of CaCO_3_ concretion, the distribution of Ca^2+^ ion c(r) quickly relaxes to the steady-state distribution given by^33^;

1 $${\rm{c}}({\rm{r}})={{\rm{c}}}_{{\rm{0}}}\,-\,{{\rm{c}}}_{{\rm{0}}}{\rm{R}}/{\rm{r}}$$

where c_0_ is the concentration of Ca^2+^ ion far from the concretion. Precipitation of CaCO_3_ proceeds if c(r) exceeds a threshold c_c_, which is determined by the solubility product.

Equating Eq. (1) and c_c_, the location of the outer edge of precipitation r_c_ is given by;

2 $${{\rm{r}}}_{{\rm{c}}}={{\rm{Rc}}}_{{\rm{0}}}/{{\rm{c}}}_{{\rm{0}}}-{{\rm{c}}}_{{\rm{c}}}$$

Then the thickness of the reaction front, r_c_ − R is written as;

3 $${\rm{L}}={{\rm{r}}}_{{\rm{c}}}\,-\,{\rm{R}}={{\rm{Rc}}}_{{\rm{0}}}/{{\rm{c}}}_{{\rm{0}}}\,-\,{{\rm{c}}}_{{\rm{c}}}$$

Equation (3) clearly shows that the thickness of reaction front L is proportional to the size of concretion R. Because the constant b is close to unity, the constant a can be written by;

4 $$a\simeq 0.081={{\rm{c}}}_{{\rm{0}}}/{{\rm{c}}}_{{\rm{0}}}\,-\,{{\rm{c}}}_{{\rm{c}}}$$

From this formula, we can obtain the threshold concentration c_c_ = c_0_ = 0.075.

**Figure 5:**
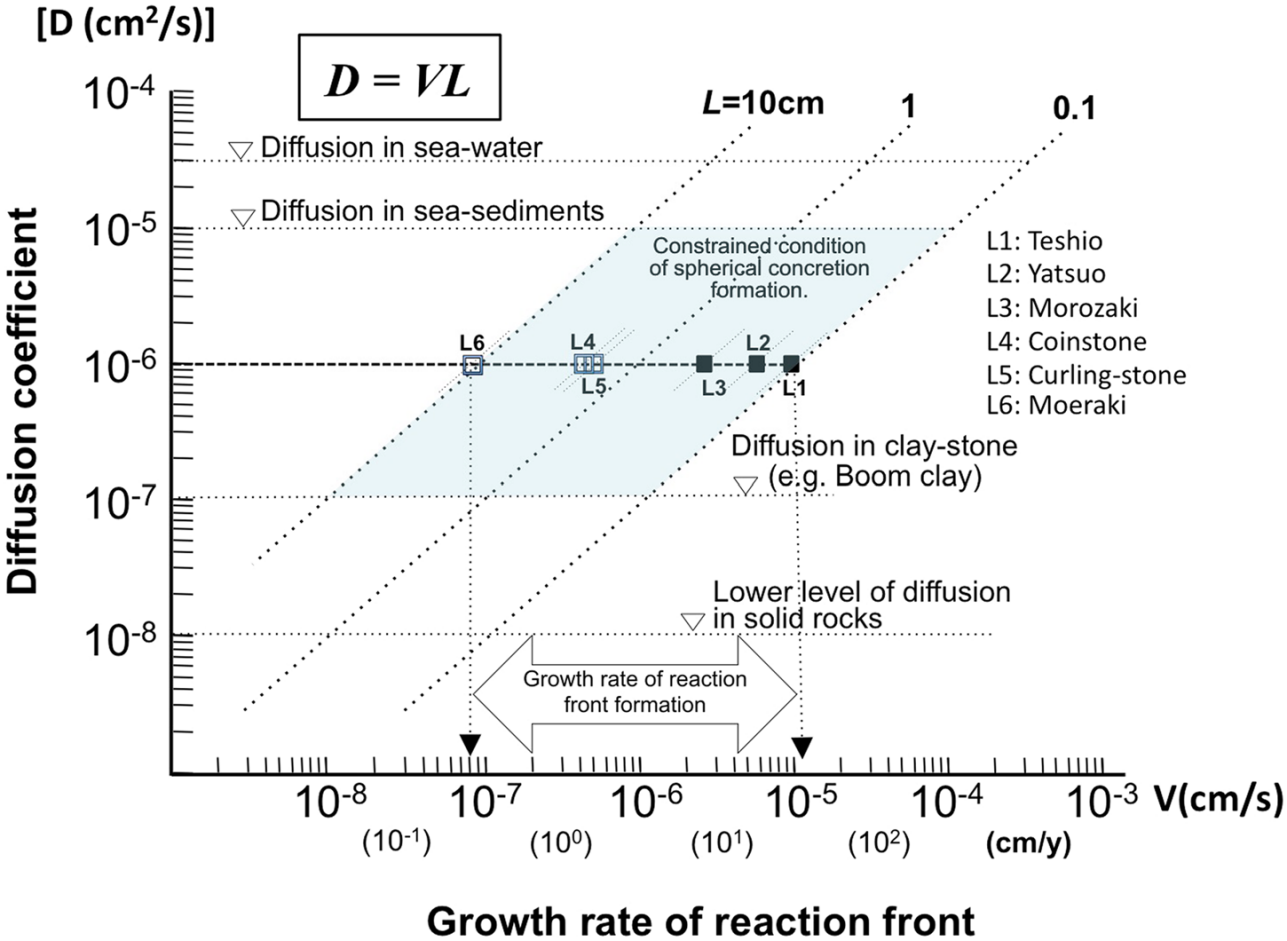
Constrained formation rate of spherical carbonate concretions. Relationship between effective diffusion coefficient (D; cm^2^/s) and growth rate of reaction front (V; cm/s) shows the narrow range of formation conditions of spherical concretions. Taking an effective diffusion coefficient for HCO_3_^−^ of 10^−6^ cm^2^/s, which is a mean value for similar kinds of clayey marine sediments together with the observed variation in reaction front thickness in small (mean values L1 to L3) to gigantic concretions (estimated values L4 to L6) shows that the growth rates of the spherical concretions was between 10^−5^ and 10^−7^ cm/s and that the concretions formed very rapidly early in diagenesis.

The correlation suggests that all examined concretions were formed under similar, relatively stable sedimentary environments in which solute transport was diffusive until formation of the concretion was completed. The relation described here between values for ‘L’ and diameters ‘R’ of concretions shows that a sharp interface is only developed when diffusion occurs in combination with rapid carbonate mineral precipitation, limited by the boundary conditions shown in Fig. 5.

A ‘Diffusion-growth rate cross plot’ (Fig. 5) enables the growth rate of spherical concretions to be deduced, as bounded by the diffusion coefficients of varied sediments (D) and the width of a reaction front (L). First of all, ‘L’ observed at the margins of concretions within marine sediments in the field ranges from a few mm to about 10 cm, as shown in Fig. 4. Diffusion coefficients can be bounded by the results of published experiments in similar sediments to those in which the studied concretions formed and by the diffusivity of solutes in seawater.

Diffusion coefficients of fine to clayey sediments, which are generally deposited on continental shelf seabeds, are in the order of 10^−6^ cm^2^/s^29^. The most reliable effective HCO_3_^−^ diffusion coefficients measured in Miocene clayey formations (e.g. Boom clay in Belgium) with similar characteristics to the sediments studied here, is (6 ± 3) ×10^−7^ cm^2^/s^34^. This latter value was measured during experiments carried out *in-situ* in an underground laboratory using radio-labelled carbonate (H^14^CO_3_^−^) under strictly defined conditions to evaluate the diffusivity^35^.

The Boom Clay, is a somewhat consolidated, plastic clay sediment (“stiff clay”)^35,36^. Therefore the diffusion coefficients measured for the Boom Clay can be used to estimate the minimum growth rates of concretions in the even less well-consolidated argillaceous sediments, within which the studied concretions are interpreted to have formed (Supplementary Table 2). On the other hand, the diffusion coefficient of dissolved concretion constituents in seawater is in the order of 10^−5^ cm^2^/s^29^.

The published measured diffusion coefficients for poorly consolidated sediments and clay, combined with the diffusivities of solutes in seawater imply that the effective diffusion coefficient of the sediment during concretion growth varied between the orders of 10^−5^ to 10^−7^ cm^2^/s. By taking a mid-range diffusion coefficient of 10^−6^ cm^2^/s and the mean value of measured ‘L’, it can be estimated that the growth rate of a reaction front varied between 10^−5^ and 10^−8^ (cm/s) (Fig. 5). These values, based on ‘L’, can be used to estimate the minimum growth rate (and hence the maximum growth time) of each spherical concretion. The estimated growth rates are at least three to four orders of magnitude faster than previously estimated timescales of concretion formation^27,28^.

The distribution of ‘L’ values on the ‘Diffusion-growth rate cross plot’ also suggests that the conditions of solute transport under which the spherical concretions formed in finer marine sediments were relatively restricted and controlled by the diffusive properties of the sediments. In particular, finer or clay rich sediments are necessary to provide lower diffusivity and permeability even with high porosity, due to the high tortuosity of the pore structures^34^. Such properties will cause slow migration of the solutes and hence ensure that HCO_3_^−^ concentrations rise sufficiently within a reaction front to provide conditions suitable for precipitation of CaCO_3_. This is the reason why concretions are observed in the field to be spherical and to have sharp boundaries that clearly distinguish them from the surrounding matrix. In coarser, porous and more permeable sediments, with higher hydraulic gradients and higher permeabilities than those of the sediments for which measured ‘L’ are reported here, it is possible that concretions may still form. However, such conditions could result in transport of solutes by porewater advection. Eventually they will have more diffuse boundaries and not such an elevated concentration of CaCO_3_ due to the fast dilution of HCO_3_^−^ produced by the decomposition of organic matter. A consequence is that such ‘concretions’ will be more difficult to recognize.

## Conclusions

Based on the investigation of several spherical concretions of different ages, the general applicability of a ‘Diffusion-growth rate cross-plot’ has been demonstrated for constraining the formation conditions of isolated spherical concretions that are widely distributed in marine argillaceous rocks deposited in relatively deep (several tens of metres) seabed environments. It has been shown that spherical concretions of different ages from different localities formed under a narrow range provided by similar clay to silt facies. All analytical data shown here imply that the spherical concretions formed very rapidly, at least three to four orders of magnitude faster than previously estimated timescales. They also maintained their characteristics, with well-preserved fossils at their centres or textures indicative of the original presence of organic matter. Simple mass balance calculations also demonstrate that the carbon fixed in the carbonate concretions came predominantly from the organs of organisms inside the concretions. Spherical carbonate concretions formed in marine sediments and having reaction fronts at their outer margins with widths, ‘L’, can be generally explained by decaying organic matter producing carbonate that then diffuses from the inside to the outside of the concretion down a concentration gradient, before being precipitated as calcite in the marginal zone. The reported method for estimating the growth rates of concretions is applicable to any syn-genetic concretion within fine marine sediments that formed due to the diffusion-controlled supply of reactants in very early diagenesis.

## Methods

Spherical carbonate concretions collected from several locations in Japan have been analyzed by the following methods. First of all, the widths of reaction fronts ‘L’ were measured on polished sections extended from the centres of these concretions and into the surrounding rock matrices. The sections were used to determine the Ca profiles across the concretions and into the matrices. In each case the marginal zone of the concretion, across which ‘L’ was measured, was identified using an X-ray fluorescence analyzer (SXAM: XGT-2000V Horiba Japan) at the Department of Education, Gifu University, Gifu, Japan. SXAM intensity maps of Teshio, Yatuso and Morozaki concretions were reduced to one-dimensional element profiles in a direction perpendicular to the concentric ring patterns identified in Ca, Mn and Fe maps of the carbonate concretions, using the lamination trace technique^37^. In the measurement, a high-intensity continuous X-ray beam (Rh anode 50 kV 1 mA), 100μm in diameter, was focused with a guide tube perpendicular to the surface of a sample, which was located on a PC-controllable X-Y stage. X-ray fluorescence from the sample surface was analyzed with the hp-Si detector of an energy-dispersion spectrometer. The results showed semi-quantitatively the two-dimensional distribution of Ca, Fe and Mn elements across the whole surface of each sample.

Mineralogical compositions of all kinds of concretions were also determined with an X-ray diffractometer (XRD; Multiflex, Rigaku Co.) using crushed and powdered samples and Cu Kα radiation (the Cu being subjected to an electron beam of 40 kV/20 mA).

XRF analyses were undertaken to measure Ca and other major elements content using a Shimadzu SXF-1200 equipped with a Rh X-ray tube. Glass beads were prepared by mixing a portion of each sample, which was ignited at 950 °C to decompose carbonates, with anhydrous lithium tetraborate flux and then fusing. Measurements were calibrated with rock reference samples issued by the Geological Survey of Japan (GSJ: Geochemical Reference Sample Data Base, http://www.aist.co.jp/RIODB/db012/welcome.html).

The δ^13^C of the concretions were also examined to determine the origin of the carbon in the carbonate. Samples were each placed in a reaction container and purged by He gas. Each sample was then reacted with H_3_PO_4_ that was introduced by a syringe. The CO_2_ gas evolved in the reaction was carried into an IR-MS (Thermo Fisher DELTA V Plus) in a He carrier gas and the stable carbon isotopic ratio was measured and reported as δ^13^C_V-PDB_.

## Electronic supplementary material

Supplementary Information
