# Supplementary material for: Generalized conditions of spherical carbonate concretion formation around decaying organic matter in early diagenesis
Source: Sci Rep. 2018 Apr 20;8:6308. doi: 10.1038/s41598-018-24205-5 (PMC5910387; doi:10.1038/s41598-018-24205-5)
Supplement: Supplementary file 1 — Supplementary Information [file 41598_2018_24205_MOESM1_ESM.pdf]

Generalized conditions of spherical carbonate concretion formation around decaying organic matter in early diagenesis

Supplementary data

Hidekazu Yoshida[1*], Koshi Yamamoto[2], Masayo Minami[3], Nagayoshi Katsuta[4], Sirono Sin-ichi[2], Richard Metcalfe[5]

1) Material Research Section, Nagoya University, University Museum, Chikusa, Nagoya, Japan
*corresponding author) dora@num.nagoya-u.ac.jp
2) Graduate School of Environmental Studies, Nagoya University, Chikusa, Nagoya, Japan
3) Institute for Space-Earth Environmental Research, Nagoya University, Chikusa, Nagoya, Japan
4) Department of Education, Gifu University, Gifu, Japan
5) Quintessa Limited, The Hub, Henley-on-Thames, Oxfordshire, UK

Within this study, spherical carbonate concretions collected from several locations in Japan have been analyzed. The results have been used in conjunction with published data from carbonate concretions in other localities in order to determine generalized formation timescales and conditions for spherical concretions. Followings are shown as supplementary data.

Correlation between the width of reaction front (L) and diameter (R)

In order to identify the relationship between concretion size and the width of reaction front (L), the following numbers of each concretion are measured and shown in Table 1: Teshio (n = 20), Yatsuo (n = 24), Morozaki (n = 12) and Moeraki (n = 10). Other 'L' values for larger concretions, for example concretions from the Lower Lias of Dorset, southern England (known as '*Coinstones*') [26], '*Curling Stones*' from the Upper Lias, Lower Jurassic sediments exposed on the NE coast of Yorkshire, England[15] and '*Moeraki boulders*' from New Zealand[27,28] are also shown (26,27,28; references shown in the main text).

Supplementary Table 1 Correlation between the width of reaction front (L) and diameter (R)

Teshio (n=20) (mm)		Yatsuo (n=24) (mm)		Morozaki (n=10) (mm)	
L	2R	L	2R	L	2R
1.8	10	3.3	23	5.0	48
1.8	11	2.7	22	4.7	69
1.7	9	2.6	20	4.0	35
1.7	12	2.6	26	3.8	42
1.5	12	2.5	30	3.5	50
1.3	9	2.4	23	3.5	35
1.3	10	2.3	18	3.2	45
1.3	14	2.3	20	3.3	30
1.2	12	2.3	21	2.9	33
1.1	9	2.3	22		41
1.1	11	2.1	16		
1.1	18	2.1	29		
1.0	10	2.1	30		
1.0	11	2.0	18		
1.0	12	2.0	19		
0.9	9	2.0	20		
0.9	10	2.0	20		
0.9	11	2.0	21		
0.8	10	2.0	22		
0.8	11	2.0	23		
		1.9	21		
		1.9	24		
		1.8	26		
		1.8	28		

Coinstone (mm)	
L	2R
40±10	450

Curing stone (mm)	
L	2R
30±10	500

Moeraki boulder (mm)	
L	2R
130±10	1700

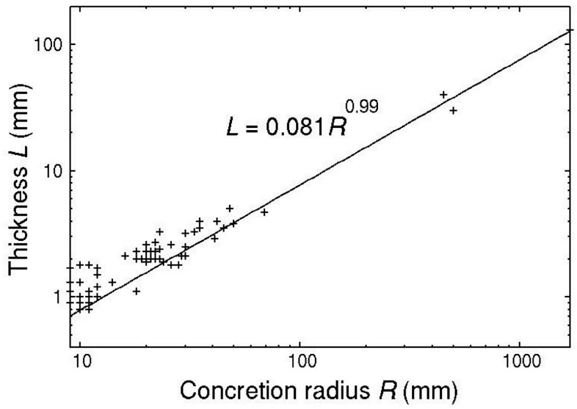

Figure 1: Least squares fitting of the data for various set of concretion radius R and the thickness of reaction front L.

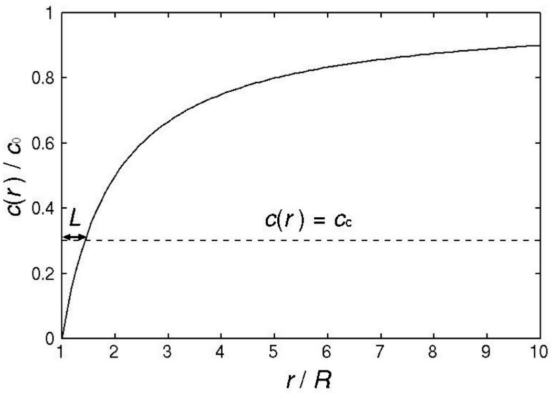

Figure 2: Concentration $c(r)=c0$ as a function of distance from the center of a concretion $r=R$. The horizontal line denotes the precipitation threshold, below which precipitation of $CaCO_3$ proceeds. The thickness of the reaction front is determined by the threshold. In this plot, $c0=cc = 0.3$ is assumed.

Diffusion coefficient of similar type of clay stone (Boom Clay)

Table 2 summarizes diffusion coefficients determined by in-situ measurement and laboratory percolation experiments on similar clay-stone i.e. Boom Clay, a Tertiary clay formation distributed in parts of western Europe (34, 35, 36; references shown in the main text). These are values for somewhat consolidated, plastic clay sediments ("stiff clay") and can be used to estimate the minimum growth rate of concretions.

Supplementary Table 2 Diffusion coefficient estimated by diffusion experiments carried out with similar type of clay-stone (Boom clay in Belgium)

	Diffusion coefficient (cm^2/s)	
**$H^{14}CO_3^-$**	$(6 \pm 3) \times 10^{-7}$	Average diffusion coefficient of Boom Clay determined by in-situ measurement (35)
	7.1×10^{-7}	Diffusion coefficient of Boom Clay determined by percolation experiments (34,36)
